# Targeted Inhibition of the miR-199a/214 Cluster by CRISPR Interference Augments the Tumor Tropism of Human Induced Pluripotent Stem Cell-Derived Neural Stem Cells under Hypoxic Condition

**DOI:** 10.1155/2016/3598542

**Published:** 2016-11-14

**Authors:** Yumei Luo, Xuehu Xu, Xiuli An, Xiaofang Sun, Shu Wang, Detu Zhu

**Affiliations:** ^1^Key Laboratory for Major Obstetric Diseases of Guangdong Province, Key Laboratory of Reproduction and Genetics of Guangdong Higher Education Institutes, The Third Affiliated Hospital of Guangzhou Medical University, Guangzhou, Guangdong 510150, China; ^2^Laboratory of Membrane Biology, New York Blood Center, New York, NY 10065, USA; ^3^Department of Gastrointestinal Surgery, The Third Affiliated Hospital of Guangzhou Medical University, Guangzhou, Guangdong 510150, China; ^4^Department of Biological Sciences, National University of Singapore, Singapore 117543; ^5^Department of Genetic Medicine, Weill Cornell Medical College, New York, NY 10065, USA

## Abstract

The human induced pluripotent stem cell (hiPSC) provides a breakthrough approach that helps overcoming ethical and allergenic challenges posed in application of neural stem cells (NSCs) in targeted cancer gene therapy. However, the tumor-tropic capacity of hiPSC-derived NSCs (hiPS-NSCs) still has much room to improve. Here we attempted to promote the tumor tropism of hiPS-NSCs by manipulating the activity of endogenous miR-199a/214 cluster that is involved in regulation of hypoxia-stimulated cell migration. We first developed a baculovirus-delivered CRISPR interference (CRISPRi) system that sterically blocked the E-box element in the promoter of the miR-199a/214 cluster with an RNA-guided catalytically dead Cas9 (dCas9). We then applied this CRISPRi system to hiPS-NSCs and successfully suppressed the expression of miR-199a-5p, miR-199a-3p, and miR-214 in the microRNA gene cluster. Meanwhile, the expression levels of their targets related to regulation of hypoxia-stimulated cell migration, such as HIF1A, MET, and MAPK1, were upregulated. Further migration assays demonstrated that the targeted inhibition of the miR-199a/214 cluster significantly enhanced the tumor tropism of hiPS-NSCs both in vitro and in vivo. These findings suggest a novel application of CRISPRi in NSC-based tumor-targeted gene therapy.

## 1. Introduction

The past decade has seen the development and application of neural stem cells (NSCs) as a novel gene delivery vector for targeted cancer gene therapy from bench to bedside [1]. NSCs are adult stem cells that hold the multipotency to differentiate into neurons, astrocytes, and oligodendrocytes, the three principal neural lineages throughout the central nervous system. When brain tumors are present, NSCs are capable of migrating through the brain parenchyma to home in on the tumor foci, including the original site and distant metastatic sites [2]. This tumor-tropic behavior has been reported to be stimulated by chemokines released from hypoxic tumors via signaling pathways such as SDF-1/CXCR4 and HGF/c-Met [3]. In animal tumor models, the innate tumor-tropic property of NSCs has been extensively exploited for targeted delivery of therapeutic genes not only to brain tumors [4, 5], but also to other disseminated metastatic solid tumors by systemic administration [6–8]. The availability of human induced pluripotent stem cell (hiPSC) technique has solved the ethical and allergic problems of NSCs in clinical applications [9, 10]. However, further in vivo study suggested that the tumor-tropic migratory capacity of human iPSC-derived NSCs (hiPS-NSCs) still has much room to improve [10, 11].

It has been described that miR-199a-5p, miR-199a-3p, and miR-214, which are coexpressed from the miR-199a/214 cluster on Chromosome 1, negatively regulate hypoxia-induced cell migration via downregulation of the HIF-1*α* and c-Met signaling [12–15]. In our previous studies, these microRNAs are among the most abundant microRNAs in hiPS-NSCs [5, 8]. Hence, we hypothesized that inhibition of the miR-199a/214 cluster may enhance the hiPS-NSCs migration towards tumors under hypoxic condition. Targeted inhibition of microRNAs can be achieved by many ways such as anti-miR oligonucleotides (AMOs), microRNA sponges, and genetic knockout; however, AMOs and microRNA sponges lack robustness as the short length (~22 nt) of mature microRNAs makes them more resistant to degradation, and genetic knockout is technically difficult and irreversible [16, 17]. Hence, more economic, precise, and efficient methods to inhibit microRNA expression are desired.

Recently, the clustered regularly interspaced short palindromic repeats (CRISPR) system has been developed as a powerful tool for targeted genome editing [18, 19]. In this system, the CRISPR-associated 9 (Cas9) nuclease is directed by a single-guide RNA (sgRNA) consisting of a 20-nt guide sequence and an auxiliary transactivating sequence via base pair complementarity to a specific genome locus to introduce double strand breaks (DSBs). The selection of sgRNA target site is restricted by the requirement of an “NGG” protospace adjacent motif (PAM) sequence next to the 3′ end. The CRISPR system can be employed to inhibit microRNA expression by destructing the loop region, the Dicer processing site, or the Drosha processing site in a specific microRNA gene [20, 21]; however, for a microRNA gene cluster, it will require to design a number of sgRNAs to target each gene. Alternatively, the microRNA gene cluster can be knocked out by the CRISPR system via genomic deletion or homologous recombination (HR) [22, 23]; however, genomic deletion requires to design 2 sgRNAs and HR requires to construct an additional donor vector.

To expand the CRISPR toolkit for transcriptional regulation, a catalytically dead Cas9 (dCas9) is created by mutating both nuclease domains of Cas9 [24]. This mutant can still be guided to specific genome loci by sgRNAs but loses DNA cleavage activity. The derivative system can be utilized to sterically occupy the promoter or gene body of a specific gene and thus block the recruitment of transcription machinery or the elongation of transcription, which is called CRISPR interference (CRISPRi). It will be more convenient to inhibit a microRNA gene cluster using CRISPRi as it requires to design only 1 sgRNA to target the promoter region of the gene cluster [20]. Moreover, the inhibitory effect by CRISPRi is reversible.

Here we have attempted to inhibit the miR-199a/214 cluster using a CRISPRi system to promote hiPS-NSC migration towards tumors under hypoxic condition. Our data showed that the CRISPRi system successfully suppressed the expression of miR-199a-5p, miR-199a-3p, and miR-214 in hiPS-NSCs and significantly enhanced their tumor tropism in vitro and in vivo.

## 2. Materials and Methods

### 2.1. Cell Reprogramming and Differentiation

A nonintegrating human iPSC (epi-hiPSC) line was generated and characterized as previously described [25]. In brief, oriP/EBNA1-based pCEP4 episomal vectors (Invitrogen, Carlsbad, CA, USA) expressing Oct4, Sox2, Klf4, L-Myc, and Lin28 were cotransfected into 1 × 10^6^ human foreskin fibroblasts (Millipore, Bedford, MA, USA). The cells were then plated onto a Matrigel (BD, Franklin Lakes, NJ, USA)-coated 10 mm dish and cultured in fibroblast medium. After 24 h, the medium was replaced by N2B27 medium supplemented with 100 ng/mL bFGF and changed every other day, up to 15 days. Then the medium was replaced by mTeSR1 medium (STEMCELL Technologies, Vancouver, Canada) and changed every day. Emerging colonies were picked onto new Matrigel-coated dishes for expansion and characterization.

NSCs were derived from the epi-hiPSC line and characterized as previously described [11]. In brief, hiPSC colonies were detached by mechanical cutting. Then, hiPSCs were dissociated into single cell suspension using TrypLE Express Dissociation Enzyme (Invitrogen) and plated onto a 0.1% gelatin-coated 6-well plate at a density of 1 × 10^6^ cells per well and cultured in NSC medium, which was a 1 : 1 mixture of DMEM/F12 (Invitrogen) supplemented with 2% B27 (Invitrogen), 2 mM L-glutamine, 50 U/mL penicillin, 50 *µ*g/mL streptomycin, 20 ng/mL EGF (Sigma-Aldrich, St Louis, MO, USA), and 20 ng/mL bFGF (Invitrogen). After 1 month of expansion, a homogeneous cell population with typical bipolar NSC morphology was achieved.

### 2.2. Cell Culture

Mouse metastatic breast cancer cell line 4T1 was purchased from American Type Culture Collection (Manassas, VA, USA). 4T1-luc cell line was purchased from Caliper (Mountain View, CA, USA). 4T1 and 4T1-luc cell lines were maintained in RPMI 1640 medium (Sigma-Aldrich) supplemented with 10% fetal bovine serum (FBS), 2 mM L-glutamine, 50 U/mL penicillin, and 50 *µ*g/mL streptomycin. For hypoxia treatment, cell cultures were incubated in a hypoxia chamber (STEMCELL Technologies) filled with an anaerobic gas mixture of 94% N_2_, 5% CO_2_, and 1% O_2_.

### 2.3. Alkaline Phosphatase (AP) Staining and Immunostaining

For AP staining, cells were fixed with 90% alcohol for 2 min and washed three times with PBS and stained with BCIP/NBT for 30 min in the darkness. For immunostaining, cells were fixed in 4.0% paraformaldehyde for 20 min, permeabilized with 0.5% Tween-20 for 30 min, incubated with primary antibody overnight, and incubated with secondary antibody (Invitrogen) for 1 hour. The nuclei were counterstained by DAPI. The cells were imaged with an inverted confocal microscope. The primary antibodies used in this study were OCT4 (1 : 500, Abcam, Cambridge, MA, USA), nestin (1 : 100, Millipore), GFAP (1 : 500, Millipore), and *β*-tubulin III (1 : 500, Millipore).

### 2.4. CRISPRi Design and Vector Construction

The fragment containing the U6-Chimeric and CBh-hSpCas9 expression cassette from the pX330 plasmid was cloned into the pFastBac1 plasmid. To create the dCas9, the D10A and H840A mutations were introduced into the RuvC1 and HNH nuclease domains of the hSpCas9 gene using the QuikChange Multi Site-Directed Mutagenesis Kit (Stratagene, La Jolla, CA, USA). The resulting plasmid was called pFB/dCas9 and used as a template to construct the baculovirus-delivered CRISPRi system.

To search an optimal sgRNA target site for CRISPRi, the sequence of the promoter region (−357 bp to −1 bp) containing an E-box element of the miR-199a/214 cluster was retrieved from the UCSC Genome Browser (http://genome.ucsc.edu/) [26]. The candidate sgRNA target sites overlaid with the E-box element (CATCTG) were screened using Optimized CRISPR Design (http://crispr.mit.edu/) [27]. Selected sgRNA target sequence was inserted into the pFB/dCas9 plasmid as previously described [28] and the resulting construct was called the CRISPRi vector in the following. The empty pFB/dCas9 plasmid was used as the control vector.

Baculoviral vectors carrying the CRISPRi system or the control construct were produced and propagated in Sf9 insect cells according to the Bac-to-Bac Baculovirus Expression system manual (Invitrogen). The hiPS-NSCs were transduced with the baculoviral vectors overnight at a multiplicity of infection of 100 plaque-forming units per cell in the NSC medium.

### 2.5. qPCR

For microRNA qPCR, small RNA was isolated using PureLink microRNA Isolation Kit (Invitrogen) and treated with TURBO DNA-free DNase (Ambion, Austin, TX, USA). Then polyA tailing and cDNA synthesis were performed using Ncode VILO microRNA cDNA Synthesis Kit (Invitrogen). The forward primers for qPCR analysis were designed based on the known mature microRNA sequence, with additional 3 “A”s at the 3′ end to improve amplification specificity (Supplementary Table 1 in Supplementary Material available online at http://dx.doi.org/10.1155/2016/3598542). The reverse primer was the Universal Primer in the EXPRESS SYBR Green microRNA qRT-PCR Kit (Invitrogen). To determine absolute copy number, a standard curve was generated using a synthetic LIN-4 RNA oligonucleotide. For mRNA qRCR, total RNA was extracted using TRIzol (Invitrogen) and first-strand cDNA was synthesized using the SuperScript III First-Strand Synthesis System for RT-PCR (Invitrogen). 1 *μ*L of cDNA reaction mix was subjected to PCR amplification using PCR SuperMix (Invitrogen). The forward and reverse primers for qPCR analysis were listed in Supplementary Table 1. GAPDH was used as the internal reference gene for relative quantification. qPCR was performed on iQ5 RT-PCR Detection System (BioRad, Berkeley, CA, USA). All reactions were run in triplicate.

### 2.6. In Vitro Boyden Chamber Cell Migration Assay

In vitro migration assays used the 4T1 cells as an attractant and were performed in Boyden chambers with the BD Falcon HTS FluoroBlok 24-well Multiwell Insert System. 4T1 cells were seeded into a 24-well companion plates in Opti-MEM (Invitrogen) at a density of 2.5 × 10^5^ cells per well. Only DMEM was used in the wells of the blank group. HiPS-NSCs were labeled with Calcein-AM (Invitrogen). The labeled hiPS-NSCs were suspended in Opti-MEM and seeded into the Boyden chamber transwell inserts at a density of 5 × 10^4^ cells per insert. After 24 h of culture at 37°C under normoxic or hypoxic conditions, the fluorescent hiPS-NSCs on the bottom side of the inserts (represent migrating cells) were counted and the migration rate was calculated. All experiments were conducted in 6 replicates.

### 2.7. Animal Experiment

All animal work was done in accordance with a protocol approved by the Institutional Animal Care and Use Committee. To access the in vivo tumor tropism of the hiPS-NSCs, a mouse model of breast cancer lung metastasis was established as previously described [29]. Six female athymic nude BALB/c mice (weight 20 g, age 6–8 weeks) were used for the assay. For lung metastasis formation, 1 × 10^5^ 4T1-luc cells in 200 *µ*L PBS per animal were injected into the mice through the tail vein. One week after the tumor inoculation, the mice were divided into 2 groups randomly. The hiPS-NSCs transduced with the CRISPRi or control baculoviral vectors were labeled with 5 *µ*g/mL of DiR (Caliper) overnight, followed by washing with PBS three times. The labeled cells, 1 × 10^6^ in 200 *µ*L PBS per animal, were injected into mice through the tail vein. The next day, luminescent signals of 4T1-luc cells were acquired using the IVIS imaging system with an emission filter of 560 nm (Caliper) after intraperitoneal injection of D-luciferin (100 mg/kg, Promega, Madison, WI, USA); fluorescent signals of DiR-labeled hiPS-NSCs were acquired using the same system with a cool CCD camera and an ICG filter (Caliper). To evaluate the in vivo DiR-labeled hiPS-NSC distribution, ex vivo imaging was performed for 9 organs: lung, liver, spleen, kidney, heart, brain, stomach, spinal cord, and femur. Images and measurements of luminescent and fluorescent signals were acquired and analyzed using the Living Image 3.2 Software (Caliper).

### 2.8. Statistical Analysis

All data are represented as mean ± SD. The statistical significance of differences was determined by paired, two-tailed Student's* t*-test. A *P* value of <0.05 was considered to be statistically significant.

## 3. Results

### 3.1. Generation and Characterization of Epi-hiPSCs and NSCs

**Figure 1:**
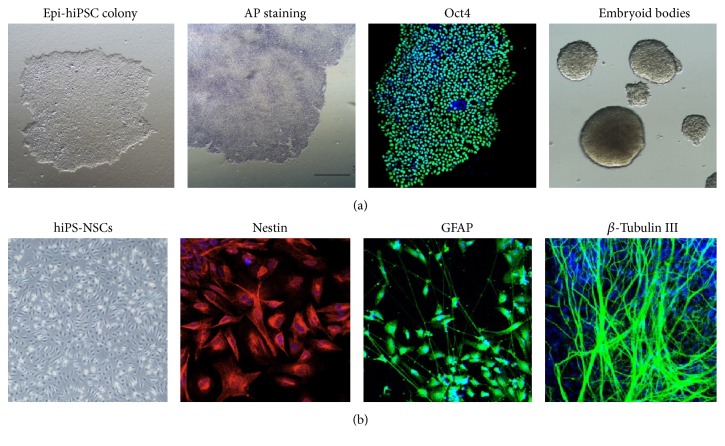
Deviation of NSCs from an epi-hiPSC line. (a) Characterization of an epi-hiPSC line generated with nonintegrating episomal vectors. From left to right: colony morphology, AP staining, immunostaining of Oct4, and embryoid body differentiation. (b) Characterization and in vitro neural differentiation of hiPS-NSCs. From left to right: bipolar cell morphology, immunostaining of nestin (NSC early stage marker), GFAP (astrocyte marker), and *β*-tubulin III (neuron marker).

An epi-hiPSC line was created from foreskin fibroblasts by electroporation of episomal vectors expressing reprogramming factors Oct4, Sox2, Klf4, L-Myc, and Lin28. The epi-hiPSC line was characterized by AP staining, immunostaining for the pluripotency marker Oct4, and embryoid body differentiation (Figure 1(a)). Then NSCs were derived from the above epi-hiPSCs by a simple adherent monolayer culture method and characterized by immunostaining for nestin, an early-stage marker of NSCs (Figure 1(b)). After sequential withdrawal of bFGF and EGF, they generated a mixed population of glial fibrillary acidic protein- (GFAP-) positive glial cells and *β*-III tubulin-positive neurons (Figure 1(b)). Thus, the epi-hiPSC-derived NSCs held the multipotency to differentiate into glial cells and neurons.

### 3.2. Targeted Inhibition of the MiR-199a/214 Cluster by a CRISPRi System in NSCs

**Figure 2:**
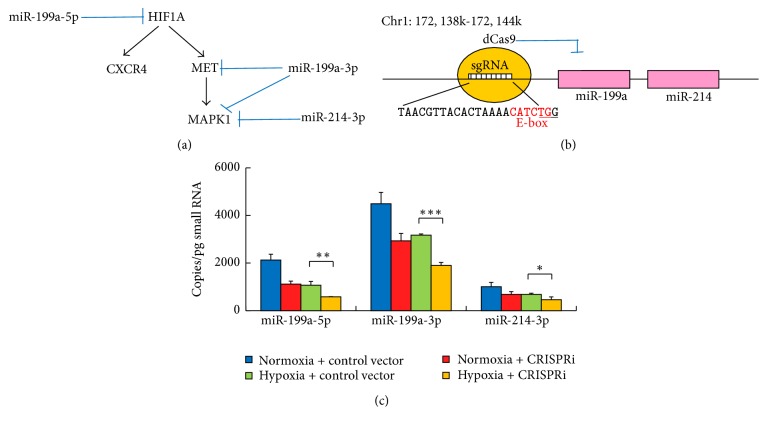
Targeted inhibition of miR-199a/214 cluster using a CRISPRi system in NSCs. (a) Diagram showing miR-199a-5p, miR-199a-3p, and miR-214 of the miR-199a/214 cluster are targeting multiple important components in the signaling pathways that mediate hypoxia-induced migration of NSCs. (b) Schematic representation showing blockage of transcription of the miR-199a/214 cluster using a dCas9 targeting the E-box in the promoter region. The sequence of the gRNA target site is shown and the PAM sequence is underlined. The E-box sequence is highlighted in red. (c) Absolute expression levels of miR-199a-5p, miR-199a-3p, and miR-214 in NSCs are quantified by qPCR (*n* = 3). Error bars: SD. ^*∗*^ *P* < 0.05, ^*∗∗*^ *P* < 0.01, and ^*∗∗∗*^ *P* < 0.001.

In the view that miR-199a-5p, miR-199a-3p, and miR-214 of the miR-199a/214 cluster are targeting the HIF-1*α* and c-Met signaling pathways that play an essential role in hypoxia-induced cell migration (Figure 2(a)) [12–15], we attempted to develop a CRISPRi system to inhibit the expression of this microRNA gene cluster. According to literature, the region between −357 bp and −1 bp of the promoter, containing an E-box element (CATCTG), is necessary to drive the transcription of the miR-199a/214 cluster [30]; hence, the sequence of this region was retrieved and screened for candidate sgRNA target sites overlaid with the E-box element. Only 1 candidate was found: “TAACGTTACACTAAAACATCTGG” at position −344 (Figure 2(b)). Since previous studies showed that the CRISPR system potentially tolerated 1–3 target mismatches [31, 32], we further looked into its off-targets with 3 mismatches or less. We found that the candidate had only 2 off-targets with 3 mismatches and these off-targets were not close to any protein coding region or transcription factor binding site (at least 300 bp up- and downstream). Therefore, the candidate was selected as the CRISPRi target.

Baculovirus was employed to efficiently deliver the CRISPRi system targeting the E-box element or the control vector into hiPS-NSCs. MicroRNA qPCR was performed to access the absolute expression levels of miR-199a-5p, miR-199a-3p, and miR-214 in hiPS-NSCs transduced with the CRISPRi system and under hypoxic condition (Figure 2(c)). Consistent with our previous studies [5, 8], the expression levels of miR-199a-5p, miR-199a-3p, and miR-214 in hiPS-NSCs were very high under normoxic condition but downregulated by 30%–50% under hypoxic condition. The CRISPRi system alone could downregulate the microRNA expression levels by 32%–48% in hiPS-NSCs under normoxic condition. Impressively, the CRISPRi system further reduced the microRNA levels by another 33%–45% under hypoxic condition. Therefore, our CRISPRi system targeting the E-box element in the promoter of the miR-199a/214 cluster successfully inhibited miR-199a-5p, miR-199a-3p, and miR-214 expression in hiPS-NSCs under hypoxic condition.

### 3.3. Inhibition of the MiR-199a/214 Cluster Promotes NSC Tumor Tropism In Vitro

**Figure 3:**
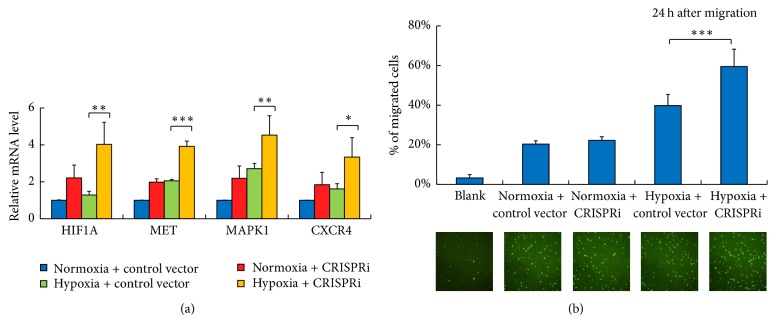
Inhibition of the miR-199a/214 cluster derepresses hypoxia-related targets and promotes NSC tumor tropism in vitro. (a) Relative expression levels of HIF1A, MET, MAPK1, and CXCR4 in NSCs are quantified by qPCR (*n* = 3). Error bars: SD. ^*∗*^ *P* < 0.05, ^*∗∗*^ *P* < 0.01, and ^*∗∗∗*^ *P* < 0.001. (b) In vitro migration of NSCs in Boyden chamber. Only DMEM is in lower chambers of the blank group. 4T1 cells are seeded in lower chambers of other groups as attractants. NSCs are transduced with baculovirus and stained with calcein-AM and seeded in the upper chamber. After 24 h, the labelled NSCs which migrated toward the lower chamber were evaluated (*n* = 6). Top: percentage of migrated NSCs. Bottom: fluorescence images showing the migration of NSCs toward the lower chambers. Error bars: SD. ^*∗∗∗*^ *P* < 0.001.

We then further investigated whether the inhibition of miR-199a/214 cluster could derepress the hypoxia-related signaling pathways and promote hiPS-NSC migration under hypoxic condition. The mRNA expression levels of the reported microRNA targets, HIF1A, MET, and MAPK1, were measured by qPCR. The CXCR4 was included as it was another important target of HIF-1*α* for hypoxia-induced cell migration towards tumors (Figure 3(a)). The results showed that, under normoxic condition, these target genes were upregulated 1.8–2.2-fold after inhibition of the miR-199a/214 cluster in hiPS-NSCs. Under hypoxic condition, the expression levels of these genes were elevated merely 1.2–2.7-fold but 3.3–4.5-fold after inhibition of the miR-199a/214 cluster.

Next, the in vitro migratory capacity of hiPS-NSCs towards 4T1, a mouse metastatic breast cancer cell line, was evaluated by Boyden chamber cell migration assays (Figure 3(b)). The results showed that, under normoxic condition, the inhibition of miR-199a/214 cluster did not increase the migration ratio of hiPS-NSCs towards 4T1 cells. This is possibly due to insufficient chemokines release from the cancer cells under normoxic condition. Under hypoxic condition, the inhibition of miR-199a/214 cluster promoted the migration ratio of hiPS-NSCs towards 4T1 cells from 40% to 60%. Taken together, our results demonstrated that the inhibition of miR-199a/214 cluster by CRISPRi could significantly improve the tumor tropism of hiPS-NSCs in vitro under hypoxic condition.

### 3.4. Inhibition of the MiR-199a/214 Cluster Enhances NSC Tumor Tropism In Vivo

**Figure 4:**
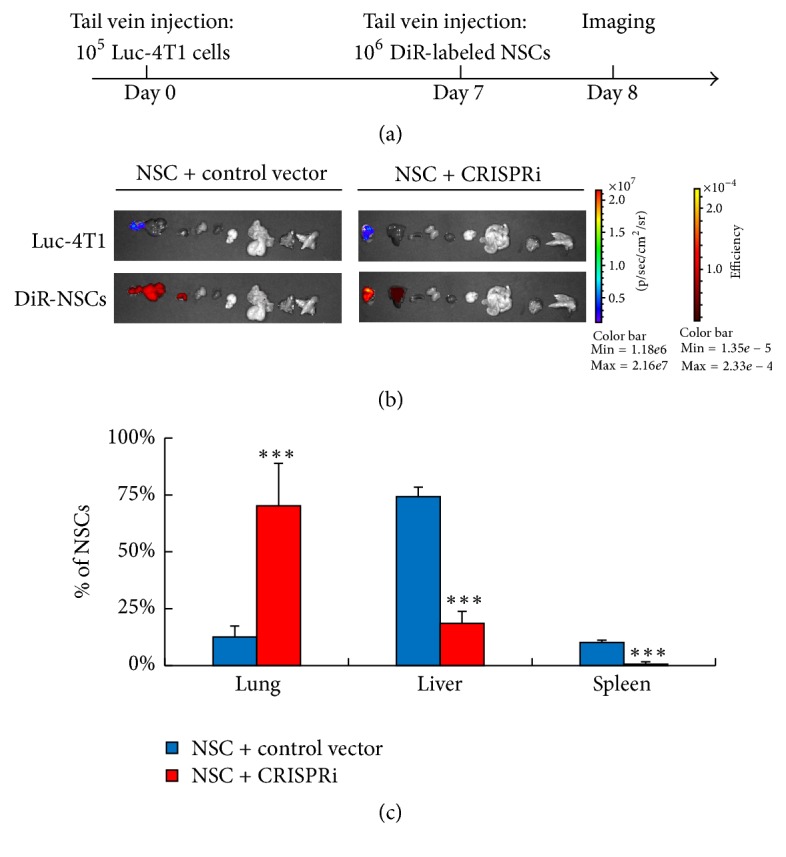
Inhibition of the miR-199a/214 cluster enhances NSC tumor tropism in vivo. (a) Diagram showing the protocol of NSC in vivo tumor tropism assay. (b) Representative ex vivo organs images showing the tumor tropism of NSCs in mice at day 8. The luminescent images (upper panels) show luc-4T1 tumor metastases in the lung. The fluorescent images (lower panels) show the organ distribution of NSCs. The organs shown in each panel from left to right: lung, liver, spleen, kidney, heart, brain, stomach, spinal cord, and femur. (c) Histogram showing the percentages of NSCs distributed in the lungs, the livers, and the spleens (*n* = 3). Error bars: SD. ^*∗∗∗*^ *P* < 0.001.

To further examine whether inhibition of the miR-199a/214 cluster could improve the hiPS-NSC tumor tropism in vivo, an animal study was performed using a mouse model of breast cancer lung metastasis. In brief, 4T1-luc metastatic breast cancer cells were inoculated into the BALB/c mice via tail vein injection. After 1 week, lung metastases were established and the mice were divided into 2 groups randomly. Then hiPS-NSCs transduced with the CRISPRi system or the control vector were labeled by DiR and injected into the mice through the tail vein, respectively. The next day, whole-body luminescent and fluorescent images were taken to confirm the presence of 4T1-luc tumors and DiR-labeled hiPS-NSCs (Figure 4(a), Supplementary Figure 1). Then, ex vivo organ imaging was performed to investigate the in vivo distribution of the hiPS-NSCs. The luminescent and fluorescent signals in 9 organs, including lung, liver, spleen, kidney, heart, brain, stomach, spine, and femur, were acquired and quantified (Figure 4(b), Supplementary Figure 2). The luminescent signals confirmed that 4T1-luc tumor metastases were established in the lung. The fluorescent signals showed that the hiPS-NSCs in both groups could home in on the lung metastases. However, in the control group, only around 12% of hiPS-NSCs were in the lung, but about 75% and 10% of cells were distracted to the liver and the spleen. Meanwhile, in the CRISPRi group, over 70% of hiPS-NSCs migrated to the lung and only around 18% of cells went to the liver (Figure 4(c), Supplementary Figure 3). In summary, the inhibition of the miR-199a/214 by CRISPRi significantly augments the tumor tropism of hiPS-NSCs in vivo.

## 4. Discussion

The poor prognosis of malignant tumors is largely due to the ability of these tumors to infiltrate in the human body and the ineffectiveness of conventional therapies, such as surgery and radio- and chemotherapy, to eliminate disseminated tumors. Apparently, there is an urgent need to develop novel therapeutic approaches to overcome the limitations of current cancer therapies. NSCs are able to home in on not only the brain tumors but also solid tumors of a nonneural origin [2, 11]. In animal tumor models, the tumor tropism of NSCs has been extensively explored for targeted delivery of anticancer agents to both original tumor masses and distant tumor metastases [6, 7]. A phase 1 clinical trial using a human NSC line to deliver a suicide gene into recurrent glioblastoma multiforme is currently ongoing at the City of Hope Medical Center in Duarte, California [1].

The great potential of NSCs in cancer gene therapy underscores the importance of a robust, reliable source for the large scale, standardized production of human NSCs that meet the requirements of good clinical practice. The availability of hiPSC technique has provided an accessible, stable source to produce unlimited amounts of NSCs for cell therapies [33, 34]. Also, the use of hiPSCs bypasses the ethical concern on human embryonic stem cells [35] and the safety issue of immune rejection by allogeneic transplantation. Although the potential clinical application of hiPSCs is attractive, there are still many safety issues on the cellular reprograming methods. The conventional lentiviral and retroviral vectors are able to deliver the reprogramming factor efficiently in most human cell types [8, 10]; however, the integration of viral genomes into host genomes may introduce unexpected mutations and thus is not suited for clinical use. To circumvent this problem, here we use episomal vectors to generate a nonintegrating epi-hiPSC line. The NSCs derived from this epi-hiPSC line also have good tumor tropism and can be genetically manipulated by baculovirus.

To our knowledge, this is the first attempt to use baculovirus to deliver a CRISPRi system into NSCs. The baculovirus is a kind of insect virus but able to transduce human cells efficiently. Unlike traditionally used human viral vectors, such as retrovirus, adenovirus, and adeno-associated virus, baculovirus is inherently incapable of replicating in human cells and there is no detectable preexisting immunity to baculovirus in human; hence, it has been extensively exploited as a novel gene delivery vector for clinical applications [36, 37]. Notably, another feature of the baculoviral vector is the large cargo capacity, up to 28 kb. Considering that Cas9 gene size is relatively large (~4 kb), a larger vector will allow design of a more complicated CRISPR-based system and expansion of the CRISPR toolkits using dCas9-effector fusion proteins.

More importantly, the animal experiment using a breast cancer lung metastasis mouse model has demonstrated that targeted inhibition of the miR-199a/214 cluster by CRISPRi significantly augmented the tumor tropism of hiPS-NSCs in vivo. The tumor microenvironment is usually hypoxic due to the high proliferation rate of tumor cells. The tumor cells will release cytokines such SDF-1 and HGF to stimulate angiogenesis to support tumor growth. After intravenous injection, the NSC vectors will travel through the organs via the circulation system. They are supposed to sense the SDF-1 and HGF gradient and migrate through the venular walls into the tumor tissues. However, the SDF-1/CXCR4 and HGF/c-Met signaling pathways are negatively regulated by the miR-199a/214 cluster in NSCs. In our animal experiment, we demonstrated that, by inhibition of the miR-199a/214 cluster, a significantly higher ratio of NSC vectors migrated into the tumor tissues. In previous animal study, it has been observed that off-target therapeutic gene expression by NSC vectors would cause serious damage to healthy organs [6]; therefore, enhancement of tumor tropism is critical for a successful NSC-mediated cancer gene therapy.

## 5. Conclusion

We have developed a baculovirus-delivered CRISPRi system that specifically inhibits the miR-199a/214 cluster on Chromosome 1 by blocking the E-box in the promoter region. Targeted inhibition of the miR-199a/214 cluster in NSCs significantly improved their tumor tropism in vitro and in vivo.

## Supplementary Material

Supplementary Materials of primer sets for qPCR and full images for the animal experiment.
