# Supplementary material for: Targeted Inhibition of the miR-199a/214 Cluster by CRISPR Interference Augments the Tumor Tropism of Human Induced Pluripotent Stem Cell-Derived Neural Stem Cells under Hypoxic Condition
Source: Stem Cells Int. 2016 Nov 14;2016:3598542. doi: 10.1155/2016/3598542 (PMC5124688; doi:10.1155/2016/3598542)
Supplement: Supplementary file 1 — Supplementary Materials of primer sets for qPCR and full images for the animal experiment. [file 3598542.f1.pdf]

Supplementary Table 1. qPCR primers

Genes	Primer Sequence
hsa-miR-199a-5p	F: 5’-CCCAGTGTTCAGACTACCTGTTCAAA-3'
hsa-miR-199a-3p	F: 5’-ACAGTAGTCTGCACATTGGTTAAAA-3'
hsa-miR-214	F: 5’-ACAGCAGGCACAGACAGGCAGTAAA-3'
cel-LIN-4	F: 5’-TCCCTGAGACCTCAAGTGTGAAAA-3’
HIF1A	F: 5’-ACAGCAGCCAGACGATCATGC-3'
	R: 5’-GCAGTAGGTTTCTGCTGCCTTGT-3'
MET	F: 5’-CCAAATCTTTTATTAGTGGTGGGAGCA-3'
	R: 5’-GCTGTTGCAGGGAAGGAGTGG-3'
MAPK1	F: 5’--GCGCGGGCCCGGAGATGGTC-3'
	R: 5’-TGAAGCGCAGTAAGATTTTT-3'
CXCR4	F: 5’-CGCATCTGGAGAACCAGCGG-3'
	R: 5’-GAGGTCGGCCACTGACAGGT-3'
GAPDH	F: 5’-TGCACCACCAACTGCTTAGC-3'
	R: 5’-GGCATGGACTGTGGTCATGAG-3'

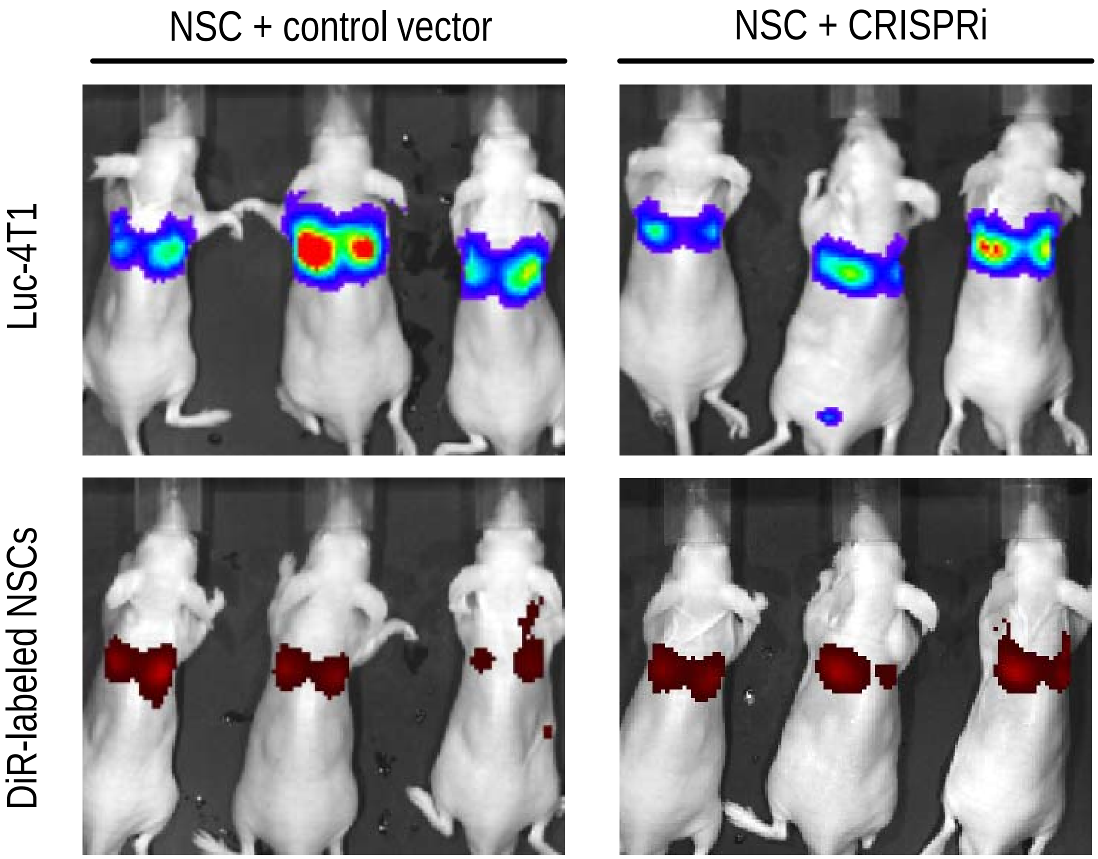

Supplemtary Figure 1: Whole body images showing the presence of luc-4T1 tumor and DiR-labeled NSCs in mice. The luminescent images show luc-4T1 tumor formation. The fluorescent images show the presence of NSCs.

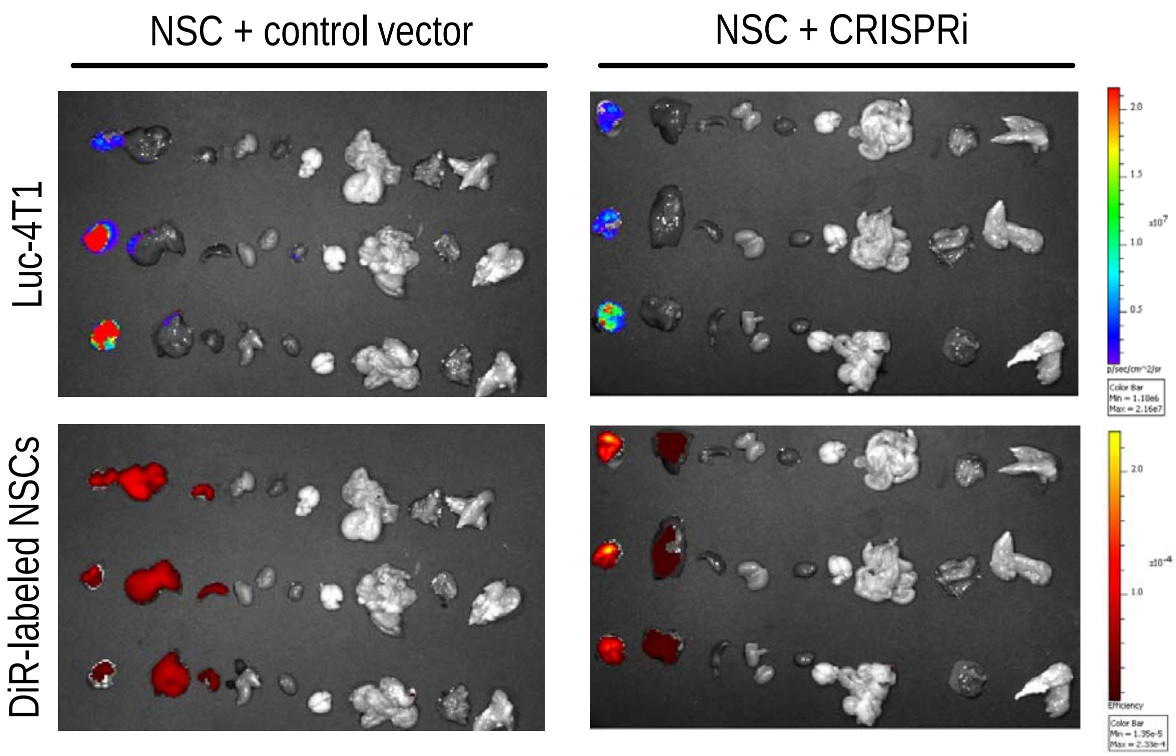

Supplemtary Figure 2: Ex vivo organs images showing the tumor tropism of NSCs in mice. The luminescent images show luc-4T1 tumor metastases in the lung. The fluorescent images show the organ distribution of NSCs. The organs shown in each panel from left to right: lung, liver, spleen, kidney, heart, brain, stomach, spinal cord and femur.

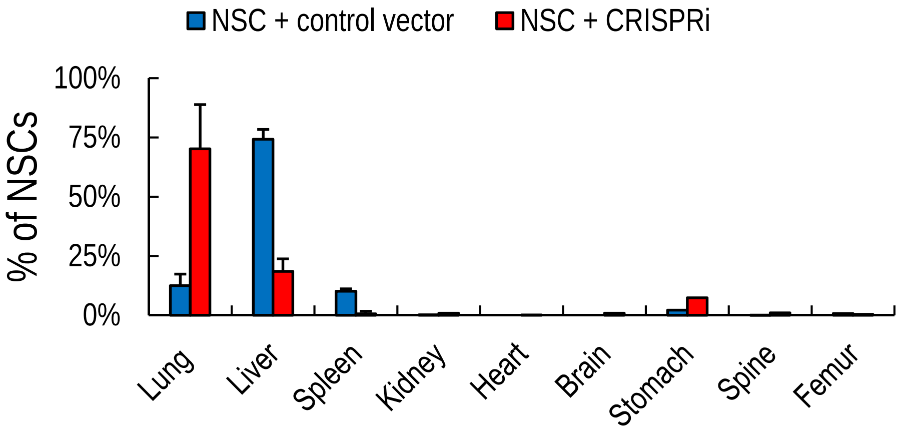

Supplemtary Figure 3: Organ distribution of NSCs. Histogram showing the percentages of NSCs distributed in different organs (n=3). Error bars: s.d.
